# Demographics and Social Factors Associated With Persistent Nonuse of Video Appointments at a Multisite Health Care Institution: Cross-Sectional Study

**DOI:** 10.2196/50572

**Published:** 2024-01-24

**Authors:** Pravesh Sharma, Celia Kamath, Tabetha A Brockman, Anne Roche, Pamela Sinicrope, Ruoxiang Jiang, Paul A Decker, Vanessa Pazdernik, Christi Patten

**Affiliations:** 1 Psychiatry and Psychology Mayo Clinic Health System Eau Claire, WI United States; 2 Quantitative Health Sciences Mayo Clinic Rochester, MN United States; 3 Health Equity and Community Engagement Research Mayo Clinic Rochester, MN United States; 4 Psychology Mayo Clinic Rochester, MN United States; 5 Psychiatry and Psychology Mayo Clinic Rochester, MN United States

**Keywords:** digital health, telemedicine, telehealth, video visits, appointments, SDoH, social determinants of health, social determinants, appointment, users, sociodemographic, prevention, discomfort, video communication, communication, willingness, mobile phone

## Abstract

**Background:**

During the COVID-19 outbreak, video appointments became a popular method for health care delivery, particularly in the early stages of the pandemic. Although Mayo Clinic aimed to reduce face-to-face (F2F) appointments to prevent the spread of the virus, some patients continued seeing their health care providers in person. In the later stages of the pandemic, many patients became comfortable with video appointments, even if they were initially hesitant. However, a subset of patients continued to avoid video appointments. It is not yet clear what sociodemographic factors may be associated with this group of patients.

**Objective:**

This cross-sectional study aimed to examine demographic and social determinant of health (SDoH) factors associated with persistent nonusers of video appointments among a sample of patients within a multistate health care organization. We also explored patient beliefs about the use of video for health care appointments.

**Methods:**

We conducted a 1-time cross-sectional paper survey, mailed between July and December 2022, of patients matching the eligibility criteria: (1) aged ≥18 years as of April 2020, (2) Mayo Clinic Midwest, Florida, or Arizona patient, (3) did not use video appointment services during April-December 2020 but attended F2F appointments in the departments of primary care and psychiatry/psychology. The survey asked patients, “Have you ever had a video appointment with a healthcare provider?” “Yes” respondents were defined as “users” (adapted to video appointments), and “no” respondents were defined as “persistent nonusers” of video appointments. We analyzed demographics, SDoH, and patient beliefs toward video appointments in 2 groups: persistent nonusers of video appointments and users. We used chi-square and 2-tailed *t* tests for analysis.

**Results:**

Our findings indicate that patients who were older, lived in rural areas, sought care at Mayo Clinic Midwest, and did not have access to the patient portal system were likely to be persistent nonusers of video appointments. Only 1 SDoH factor (not having a disability, handicap, or chronic disease) was associated with persistent nonuse of video appointments. Persistent nonusers of video appointments held personal beliefs such as discomfort with video communication, difficulty interpreting nonverbal cues, and personal preference for F2F appointments over video.

**Conclusions:**

Our study identified demographic (older age and rural residence), sociodemographic factors (not having a disability, handicap, or chronic disease), and personal beliefs associated with patients’ decisions to choose between video versus F2F appointments for health care delivery. Health care institutions should assess patients’ negative attitudes toward technology prior to introducing them to digital health care services. Failing to do so may result in its restricted usage, negative patient experience, and wasted resources. For patients who hold negative beliefs about technology but are willing to learn, a “digital health coordinator” could be assigned to assist with various digital health solutions.

## Introduction

Since the COVID-19 pandemic, video appointments have been widely implemented for remotely delivered health care [1,2]. Both prepandemic and pandemic literature suggest that video visits improve provider access to patients, reduce patient travel and wait times, and provide health care quality comparable to face-to-face (F2F) appointments [3-6]. Despite these positive associations with telemedicine, video appointments for nonemergent care have not been as widely used by patients as expected. Studies show that individuals who could not adapt to digital health care delivery have faced significant health care access barriers during and since the pandemic [7]. In the current post-COVID-19 era, digital health care services are a new standard of care [8], and patients who need to be connected to the health care system digitally but are struggling to adapt to telemedicine may experience suboptimal health care [9]. Therefore, factors associated with nonengagement with video visits, especially in patients who have *persistently not engaged* in video appointments, require further exploration. While patients’ attitudes to telemedicine, especially in the COVID-19 era [10-12], have been explored, gaps remain in understanding social and individual characteristics associated with the *persistent nonuse of video appointments* for health care.

A large body of evidence suggests that older age, low education, poor digital access (broadband [BB] internet and smart devices), [13] and personal preferences [14] are independently and interactively associated with lower engagement with digital health care [14-17]. This is ironic, given that a critical reason behind the embarkation of digital health care technology was to provide uninterrupted health care access to those who live in remote areas where access to health care providers is limited, those who experience low socioeconomic status and associated transportation challenges and those with poor mobility due to old age and other constraints [18,19]. Evidence also shows that if the individual digital barriers are addressed [20-22], people are willing to engage in technology and participate in telemedicine programs. Preliminary public and institutional efforts to mitigate patient-related barriers to telemedicine are in their infancy but may include brief verbal and printed technology instructions, digital navigation programs for those who have poor digital literacy (comfort and ease of using technology), use of public Wi-Fi and “to-go kits” (smart devices with written instructions on connecting for a visit) [18,23]. Despite nationwide initiatives that accelerated after COVID-19 to encourage patients to use digital health care, many still chose to engage in F2F appointments [14,24]. This phenomenon was observed with and without social distancing associated with the COVID-19 pandemic.

Social determinants of health (SDoH) such as economic stability, access to quality education and health care, neighborhood safety and housing, community and social contexts, and experiences of racism and discrimination [25] significantly impact people’s well-being. The adverse outcomes associated with SDoH inequitably impact marginalized groups and prevent them from accessing quality health care. SDoH not only impact how easily and efficiently people can access health care, but also how they will access it (video vs F2F vs both).

For example, evidence shows that patients who identified as Black, indigenous, or people of color, and were non-English speaking patients and lived in neighborhoods with low socioeconomic status were less likely to engage in digital health care [17,26]. However, it has not been investigated yet which SDoH factor is linked to the persistent use and nonuse of video technology for health care appointments.

This cross-sectional study aimed to examine demographic and SDoH factors associated with no video use (self-reported persistent nonusers of video appointments) among a sample of patients within a large multistate health care organization. We also explored patient beliefs about the use of video for health care appointments. We hypothesized that certain demographic factors, including older age, being a woman, low education, rural residence, and SDoH, such as financial constraints and limited transportation options, may be associated with the persistent nonuse of video appointments.

## Methods

### Setting

Mayo Clinic consists of a large academic medical center and associated health system spanning the United States in 5 states (Minnesota, Wisconsin, Iowa, Florida, and Arizona). Mayo Clinic’s main campuses are located in Rochester, Minnesota; Phoenix, Arizona; and Jacksonville, Florida. Mayo Clinic Health System (MCHS) consists of clinics, hospitals, and other health care facilities in 4 regions in southern Minnesota, western Wisconsin, and northern Iowa. The Mayo Clinic Midwest (MN, WI, and IA) serves patients that are predominantly White, older people, and living in rural areas. In contrast, Mayo Clinic in Florida and Arizona serve a more diverse patient population.

### Ethics Approval

This study was approved by the Mayo Clinic Institutional Review Board (21-004523).

### Study Overview and Design

During earlier phases of COVID-19, in early 2020, the US government and the Centers for Disease Control and Prevention recommended social distancing measures, including stay-at-home orders and video appointments with health care providers [27]. Despite Mayo Clinic’s attempts to minimize F2F appointments to prevent the spread of the virus, many patients requested F2F appointments with their health providers. We were therefore interested in examining whether demographic and SDoH, including area-based metrics (where patients live), were associated with F2F visits. This study used a cross-sectional design with data collected from a 1-time survey administered to Mayo Clinic and MCHS patients.

### Survey Instrument and Measures

The survey was designed using results from a prior qualitative study detailed elsewhere [14]. Guided by the qualitative results and informed by a scoping literature review, the survey items were developed to address existing gaps in the literature. The finalized paper survey was pretested with study staff with an estimated 10-15 minutes to complete. The survey included 21 items querying patient’s digital access such as BB internet connection and smart devices, digital literacy (the ease and comfort of using digital technology), use of the patient portal (Mayo Clinic patient online messaging system), use of video appointments, attitudes, and beliefs toward F2F versus video appointments and barriers to engaging in video appointments. The SDoH-related questions included in our survey were adapted from the Social Needs Screening Tool [28] (Multimedia Appendix 1).

Demographic characteristics (age, gender, and race or ethnicity), education status (highest during this study’s period), patient portal status (yes or no), and residence zip codes were extracted from the electronic health record (EHR). Rurality was ascertained from patient zip codes to identify corresponding rural-urban commuting area (RUCA) codes based on the University of Washington classification C method classification [29].

The *dependent (outcome) variable* was a dichotomous response (yes or no) to the question, “Have you ever had a video appointment with a healthcare provider?”

### Data Collection or Procedure

We extracted data from the EHR of adult patients with this study’s eligibility of (1) being aged ≥18 years *as of April 2020*, (2) being a Mayo Clinic Midwest (Rochester or MCHS), Florida or Arizona patient, (3) not using video appointment services during the time frame of April-December 2020 but attending F2F appointments for nonemergent outpatient clinical care in the departments of primary care and psychiatry/psychology.

The Mayo Clinic Survey Research Center mailed eligible patients a survey in a prelabeled return envelope in early July 2022. By that time, a significant number of patients were oriented and made aware of video appointment procedures through self-learning and efforts by our health care institutions. Thus, the following survey item: “Have you ever had a video appointment with a healthcare provider?” with dichotomous responses “Yes/No,” provided valuable cross-sectional information distinguishing patients in this cohort in terms of their ability to adapt or not to evolving remote health care delivery appointments through video appointments for nonemergent care after April 2020. The respondents who marked “no” were defined as “persistent nonusers.” In contrast, those who responded “yes” were defined as “users” who, despite not having used video appointments between April and December 2020, adapted to the changing digital landscape, using them later.

Surveys were mailed to eligible patients stratified by departmental visit type (psychiatry/psychology versus primary care), demographic characteristics (gender, race, and Mayo Clinic location), and if the patient has an active patient portal account. The Survey Research Center mailed reminder letters to nonresponders in August 2022 and then conducted phone call reminders to nonresponders in October-December 2022. Survey participation was closed in January 2023. Survey respondents received a sheet of forever stamps valued at US $5.

### Statistical Analysis

Demographics, SDoH, and patient beliefs about video encounters were compared across groups, persistent nonusers of video appointments, and users groups, using the chi-square (exact) test for categorical variables and the 2-sample *t* test (rank sum) for continuous variables. In all cases, *P* values <.05 were considered statistically significant.

## Results

### Overview

Respondent sociodemographic characteristics (N=321) are described in Table 1 overall and by use of video appointments. The survey response rate was 11% (321/3000). In the total respondent sample, 172 (54%) were women, 217 (68%) were White, 169 (53%) had bachelor’s or higher education degrees (persistent nonusers vs users; 84, 52.5% vs 85, 52.8%), and 282 (88%) were urban dwelling (persistent nonusers vs users; 133, 83.1% vs 149, 92.5%; *P*=.01). In addition, 266 ( 83%) had access to an online patient portal account (persistent nonusers vs users; 122, 76.2% vs 144, 89.4%; *P*=.002).

**Table 1:** Demographic factors associated with the using and not using video appointments.

Demographics		Total (N=321), n (%)	Persistent nonusers of video (n=160), n (%)	Users of video (n=161), n (%)	*P* value	
**Age (y)**						.001^a^
	Mean (SD)	57.440 (16.400)	60.434 (16.849)	54.465 (15.425)		
	Range	18.111-90.010	18.111-90.010	21.791-86.867		
**Gender**						.05^b^
	Women	172 (53.6)	77 (48.1)	95 (59)		
	Men	149 (46.4)	83 (51.9)	66 (41)		
**Specialty**						>.99^c^
	Community medicine	309 (96.3)	154 (96.2)	155 (96.3)		
	General internal medicine	6 (1.9)	3 (1.9)	3 (1.9)		
	Psychiatry and psychology	6 (1.9)	3 (1.9)	3 (1.9)		
**Site**						.02^b^
	Arizona	134 (41.7)	67 (41.9)	67 (41.6)		
	Florida	96 (29.9)	38 (23.8)	58 (36)		
	Mayo Clinic Midwest	91 (28.3)	55 (34.4)	36 (22.4)		
**Race**						.38^b^
	Non-White	89 (27.7)	47 (29.4)	42 (26.1)		
	Unknown	15 (4.7)	5 (3.1)	10 (6.2)		
	White	217 (67.6)	108 (67.5)	109 (67.7)		
**Education**						.54^b^
	Less than equal to 12th grade	30 (9.3)	16 (10)	14 (8.7)		
	Some college, no degree	22 (6.9)	9 (5.6)	13 (8.1)		
	Associate degree	36 (11.2)	18 (11.2)	18 (11.2)		
	Bachelors	84 (26.2)	36 (22.5)	48 (29.8)		
	Higher education	85 (26.5)	48 (30)	37 (23)		
	Decline to answer	64 (19.9)	33 (20.6)	31 (19.3)		
**Marital status**						.36^c^
	Married	238 (74.1)	117 (73.1)	121 (75.2)		
	Single, separate, divorced, or widowed	71 (22.1)	39 (24.4)	32 (19.9)		
	Unknown or chose “N”	12 (3.7)	4 (2.5)	8 (5)		
**Rural versus urban**						.01^b^
	Rural	39 (12.1)	27 (16.9)	12 (7.5)		
	Urban	282 (87.9)	133 (83.1)	149 (92.5)		
**Portal (online patient messaging system)**						.002^b^
	No	55 (17.1)	38 (23.8)	17 (10.6)		
	Yes	266 (82.9)	122 (76.2)	144 (89.4)		

^a^Two-sample 2-tailed *t* test.

^b^Chi-squared test.

^c^Fisher exact test.

### Demographic Correlates to Persistent Nonuse of Video Appointments

Persistent nonusers of video appointments were older than users (*P*=.001). In addition, patients living in rural residences (*P*=.01) were more likely to be persistent nonusers of video appointments. Other demographic factors, such as gender, education, and race, were not significantly different between persistent nonusers and users of video appointments (Table 1).

### Institution Site Correlates to Persistent Nonuse of Video Appointments

Patients who sought care at Mayo Clinic Midwest, comprising Mayo Clinic, Rochester, and MCHS, were more likely to be persistent nonusers of video appointments (*P*=.02; Table 1).

### Social Correlates to Persistent Nonuse of Video Appointments

Patients without any disability, handicap, or chronic disease were more likely to be persistent nonusers of video appointments than users (*P*=.01; Table 2). Other SDoH-related factors were not statistically significant.

**Table 2:** Social determinant of health factors associated with using and not using video appointments.

Variable			Total (N=321)		Persistent nonusers of video (n=160)		Users of video (n=161)		*P* value^a^	
**Within the past 12 months, did you worry that your food would run out before you got money to buy more?**										.12
	Missing	1		1		0				
	No, n (%)	311 (97.2)		157 (98.7)		154 (95.7)				
	Yes, n (%)	7 (2.2)		1 (0.6)		6 (3.7)				
	Prefer not to answer, n (%)	2 (0.6)		1 (0.6)		1 (0.6)				
**Within the past 12 months, did the food you bought just not last, and you did not have money to get more?**										.45
	Missing	2		2		0				
	No, n (%)	311 (97.5)		156 (98.7)		155 (96.3)				
	Yes, n (%)	7 (2.2)		2 (1.3)		5 (3.1)				
	Prefer not to answer, n (%)	1 (0.3)		0 (0)		1 (0.6)				
**Do you have housing?**										.10
	Missing	1		1		0				
	No, n (%)	9 (2.8)		7 (4.4)		2 (1.2)				
	Yes, n (%)	310 (96.9)		152 (95.6)		158 (98.1)				
	Prefer not to answer, n (%)	1 (0.3)		0 (0)		1 (0.6)				
**Are you worried about losing your housing?**										>.99
	Missing	1		1		0				
	No, n (%)	309 (96.6)		154 (96.9)		155 (96.3)				
	Yes, n (%)	9 (2.8)		4 (2.5)		5 (3.1)				
	Prefer not to answer, n (%)	2 (0.6)		1 (0.6)		1 (0.6)				
**Within the past 12 months, have you or your family members you live with been without utilities?**										.62
	Missing	5		4		1				
	No, n (%)	311 (98.4)		155 (99.4)		156 (97.5)				
	Yes, n (%)	4 (1.3)		1 (0.6)		3 (1.9)				
	Prefer not to answer, n (%)	1 (0.3)		0 (0)		1 (0.6)				
**Within the past 12 months, lack of transportation?**										.28
	Missing	1		1		0				
	No, n (%)	312 (97.5)		154 (96.9)		158 (98.1)				
	Yes, n (%)	7 (2.2)		5 (3.1)		2 (1.2)				
	Prefer not to answer, n (%)	1 (0.3)		0 (0)		1 (0.6)				
**Within the past 12 months, did you have trouble paying your bills?**										.89
	Missing	1		1		0				
	No, n (%)	304 (95)		152 (95.6)		152 (94.4)				
	Yes, n (%)	14 (4.4)		6 (3.8)		8 (5)				
	Prefer not to answer, n (%)	2 (0.6)		1 (0.6)		1 (0.6)				
**Does any disability, handicap, or chronic disease make it difficult for you to engage in your typical activities?**										.01
	Missing	1		1		0				
	No, n (%)	279 (87.2)		147 (92.5)		132 (82)				
	Yes, n (%)	36 (11.2)		11 (6.9)		25 (15.5)				
	Prefer not to answer, n (%)	5 (1.6)		1 (0.6)		4 (2.5)				
**Are you currently working for pay?**										.61
	Missing	3		2		1				
	No, n (%)	145 (45.6)		74 (46.8)		71 (44.4)				
	Yes, n (%)	172 (54.1)		83 (52.5)		89 (55.6)				
	Prefer not to answer, n (%)	1 (0.3)		1 (0.6)		0 (0)				

^a^Fisher exact test.

### Video Encounter-Related Beliefs Correlate to Persistent Nonusers of Video Appointments

Scenario 1: “Imagine you are having a video appointment with a Mayo Clinic doctor for a general medicine health check-up that does not require any procedures or exams. Further, imagine you have seen this doctor before for a face-to-face or in-person visit.” A significantly lower proportion of persistent nonusers responded “agree,” while a significantly higher proportion of persistent nonusers of video appointments responded “somewhat disagree” or “disagree,” respectively, to the following statements in response to this scenario: “I am confident my doctor would be able to address any medical concerns effectively” (*P*=.047), “I am confident I would be able to express all my concerns clearly” (*P*=.04) and “I am confident I would feel comfortable enough to talk openly” (*P*<.001) compared to users (Table 3). No other responses were statistically significantly associated with the comparison groups.

**Table 3:** Patients’ beliefs about video encounters and their association with the use of video appointments.

Variable			Total (N=321)	Persistent nonusers of video (n=160)	Users of video (n=161)	*P* value	
**Scenario #1: “Imagine you are having a video appointment with a Mayo Clinic doctor for a general medicine health check-up that does not require any procedures or exams. Further, imagine you have seen this doctor before for a face-to-face or in-person visit.”**							
	**I am confident my doctor would be able to address any medical concerns effectively**						.047^a^
		N=miss	4	3	1		
		1=agree, n (%)	186 (58.7)	80 (51)	106 (66.2)		
		2=somewhat agree, n (%)	91 (28.7)	52 (33.1)	39 (24.4)		
		3=somewhat disagree, n (%)	19 (6)	12 (7.6)	7 (4.4)		
		4=disagree, n (%)	21 (6.6)	13 (8.3)	8 (5)		
	**I am confident I would be able to express all my concerns clearly**						.04^a^
		N=miss	5	3	2		
		1=agree, n (%)	222 (70.3)	99 (63.1)	123 (77.4)		
		2=somewhat agree, n (%)	64 (20.3)	41 (26.1)	23 (14.5)		
		3=somewhat disagree, n (%)	16 (5.1)	9 (5.7)	7 (4.4)		
		4=disagree, n (%)	14 (4.4)	8 (5.1)	6 (3.8)		
	**I am confident I would feel comfortable enough to talk openly**						<.001^b^
		N=miss	6	4	2		
		1=agree, n (%)	247 (78.4)	106 (67.9)	141 (88.7)		
		2=somewhat agree, n (%)	46 (14.6)	33 (21.2)	13 (8.2)		
		3=somewhat disagree, n (%)	11 (3.5)	8 (5.1)	3 (1.9)		
		4=disagree, n (%)	11 (3.5)	9 (5.8)	2 (1.3)		
	**I feel video appointments should cost the same and are of equal value to face-to-face appointments**						.09^a^
		N=miss	7	4	3		
		1=agree, n (%)	66 (21)	25 (16)	41 (25.9)		
		2=somewhat agree, n (%)	80 (25.5)	45 (28.8)	35 (22.2)		
		3=somewhat disagree, n (%)	91 (29)	43 (27.6)	48 (30.4)		
		4=disagree, n (%)	77 (24.5)	43 (27.6)	34 (21.5)		
**Scenario #2: Imagine you are having an appointment with a Mayo Clinic psychiatrist or psychologist that does not require any procedures or exams. Further, imagine you have seen this doctor before for a face-to-face or in-person visit.**							
	**I am confident my doctor would be able to address any medical concerns effectively**						.09^a^
		N=miss	7	4	3		
		1=agree, n (%)	179 (57)	79 (50.6)	100 (63.3)		
		2=somewhat agree, n (%)	81 (25.8)	43 (27.6)	38 (24.1)		
		3=somewhat disagree, n (%)	32 (10.2)	21 (13.5)	11 (7)		
		4=disagree, n (%)	22 (7)	13 (8.3)	9 (5.7)		
	**I am confident I would be able to express all my concerns clearly**						.03^a^
		N=miss	9	5	4		
		1=agree, n (%)	193 (61.9)	84 (54.2)	109 (69.4)		
		2=somewhat agree, n (%)	72 (23.1)	41 (26.5)	31 (19.7)		
		3=somewhat disagree, n (%)	27 (8.7)	19 (12.3)	8 (5.1)		
		4=disagree, n (%)	20 (6.4)	11 (7.1)	9 (5.7)		
	**I am confident I would feel comfortable enough to talk openly**						.001^a^
		N=miss	9	5	4		
		1=agree, n (%)	202 (64.7)	84 (54.2)	118 (75.2)		
		2=somewhat agree, n (%)	67 (21.5)	42 (27.1)	25 (15.9)		
		3=somewhat disagree, n (%)	24 (7.7)	15 (9.7)	9 (5.7)		
		4=disagree, n (%)	19 (6.1)	14 (9)	5 (3.2)		
	**I feel video appointments should cost the same and are of equal value to face-to-face appointments**						.26^a^
		N=miss	9	5	4		
		1=agree, n (%)	94 (30.1)	39 (25.2)	55 (35)		
		2=somewhat agree, n (%)	84 (26.9)	46 (29.7)	38 (24.2)		
		3=somewhat disagree, n (%)	70 (22.4)	38 (24.5)	32 (20.4)		
		4=disagree, n (%)	64 (20.5)	32 (20.6)	32 (20.4)		
**Video encounter-related beliefs not specific to any discipline**							
	**I am confident I would be able to understand when the doctor explains my symptoms or health**						.046^b^
		N=miss	7	5	2		
		1=agree, n (%)	206 (65.6)	90 (58.1)	116 (73)		
		2=somewhat agree, n (%)	79 (25.2)	47 (30.3)	32 (20.1)		
		3=somewhat disagree, n (%)	17 (5.4)	10 (6.5)	7 (4.4)		
		4=disagree, n (%)	12 (3.8)	8 (5.2)	4 (2.5)		
	**I am confident I would be able to read my doctor’s facial expressions or nonverbal cues**						.05^a^
		N=miss	6	5	1		
		1=agree, n (%)	140 (44.4)	57 (36.8)	83 (51.9)		
		2=somewhat agree, n (%)	114 (36.2)	62 (40)	52 (32.5)		
		3=somewhat disagree, n (%)	41 (13)	25 (16.1)	16 (10)		
		4=disagree, n (%)	20 (6.3)	11 (7.1)	9 (5.6)		
	**I am confident I would be able to hear my doctor clearly**						.004^b^
		N=miss	7	6	1		
		1=agree, n (%)	200 (63.7)	85 (55.2)	115 (71.9)		
		2=somewhat agree, n (%)	79 (25.2)	43 (27.9)	36 (22.5)		
		3=somewhat disagree, n (%)	22 (7)	16 (10.4)	6 (3.8)		
		4=disagree, n (%)	13 (4.1)	10 (6.5)	3 (1.9)		
	**I would enjoy connecting with my doctor as much as if the appointment were face-to-face**						.009^a^
		N=miss	4	3	1		
		1=agree, n (%)	108 (34.1)	40 (25.5)	68 (42.5)		
		2=somewhat agree, n (%)	88 (27.8)	46 (29.3)	42 (26.2)		
		3=somewhat disagree, n (%)	68 (21.5)	38 (24.2)	30 (18.8)		
		4=disagree, n (%)	53 (16.7)	33 (21)	20 (12.5)		
	**I would feel comfortable talking with a doctor I have met before in-person**						.16^b^
		N=miss	7	5	2		
		1=agree, n (%)	221 (70.4)	101 (65.2)	120 (75.5)		
		2=somewhat agree, n (%)	64 (20.4)	36 (23.2)	28 (17.6)		
		3=somewhat disagree, n (%)	15 (4.8)	8 (5.2)	7 (4.4)		
		4=disagree, n (%)	14 (4.5)	10 (6.5)	4 (2.5)		
	**I would feel comfortable talking with a doctor I have never met before in-person**						.01^a^
		N=miss	7	5	2		
		1=agree, n (%)	84 (26.8)	34 (21.9)	50 (31.4)		
		2=somewhat agree, n (%)	102 (32.5)	44 (28.4)	58 (36.5)		
		3=somewhat disagree, n (%)	76 (24.2)	44 (28.4)	32 (20.1)		
		4=disagree, n (%)	52 (16.6)	33 (21.3)	19 (11.9)		

^a^Chi-squared test.

^b^Fisher exact test.

Scenario 2: “Imagine you are having an appointment with a Mayo Clinic Psychiatrist or Psychologist that does not require any procedures or exams. Further, imagine you have seen this doctor before for a face-to-face or in-person visit.” A significantly lower proportion of persistent nonusers of video appointments responded “agree,” while a significantly higher proportion of persistent nonusers of video appointments responded “somewhat disagree” or “disagree,” respectively, to the following statements in response to this scenario: “I am confident I would be able to express all my concerns clearly” (*P*=.03), and “I am confident I would feel comfortable enough to talk openly” (*P*=.001) compared to users. No other responses were statistically significantly associated with the comparison groups.

### Video Encounter-Related Beliefs as a Correlate to Persistent Nonuse of Video Appointments

A significantly lower proportion of persistent nonusers of video appointments responded “agree,” while a significantly higher proportion of persistent nonusers of video appointments responded “somewhat disagree” or “disagree” to the following statements: “I am confident I would be able to understand when the doctor explains my symptoms/health” (*P*=.046), “I am confident I would be able to read my doctor’s facial expressions or non-verbal cues” (*P*=.05), “I am confident I would be able to hear my doctor clearly” (*P*=.004), “I would enjoy connecting with my doctor as much as if the appointment were face-to-face” (*P*=.009), and “I would feel comfortable talking with a doctor I have never met before in-person” (*P*=.01) compared to users. No other responses were statistically significantly associated with the comparison groups.

## Discussion

### Principal Findings

This cross-sectional study demonstrated demographic and SDoH factors associated with persistent nonusers of video appointments for health care in a multisite medical institution. We observed that about 50% (161 of 321) of respondents persistently have not engaged with video appointments for nonemergent primary and psychiatric care since the start of video appointments in our institution (April 2020). We further observed that patients of older age, those living in rural residences, those who sought care at Mayo Clinic Midwest and those who did not have access to the patient portal system were more likely to be persistent nonusers of video appointments. Only a single SDoH-related factor (not having a disability, handicap, or chronic disease) was associated with persistent nonuse of video appointments. We also observed that individuals held certain personal beliefs about video appointments that were associated with their decision to use versus not use video appointments for health care. The persistent nonusers of video appointments held beliefs that included being potentially uncomfortable communicating with their doctor through video, difficulty reading their doctor’s facial expressions or nonverbal cues, struggle to hear the doctor clearly, and overall better comfort with F2F appointments over video appointments.

Much evidence has demonstrated that older patients have limited engagement with telemedicine, including using video appointments for their health care needs [30-32]. Our study observed an analogous association with older age correlated with persistent nonuse of video appointments for health care. Given that few individuals in our sample experienced substantial limitations in SDoH (Table 2) and that most respondents lived in urban dwellings and had access to the online patient portal (which requires smart devices and internet BB connection), we speculate that factors other than just digital access barriers should be considered when approaching older patients for increasing digital engagement. One possible factor is limited interest in digital health care due to negative personal beliefs toward video appointments. Given that older adulthood is a period when many individuals experience a decline in physical and cognitive health and could lose interest in exploring newer concepts (technology in this case), it is essential for health care providers and health care systems to take a patient-centered approach to understand the reasoning behind an older adult patient’s preference for in-person versus video appointment and provide the appropriate support and develop barrier mitigating strategies tailored to age to engage these individuals with needed care. This study found that patients who lived in rural areas were more likely to be persistent nonvideo users. This finding has been established by many studies. A key reason for the rural-urban digital health disparity is unequal access to BB connections. Additionally, individuals living in rural areas tend to be older, have limited education, and lack the financial resources to invest in BB connections and smartphones. Overall, our research effectively collected information on demographic indicators associated with not using video appointments that parallels the geographic demographics of Mayo Clinic, Rochester and various MCHS locations in rural areas with mostly older White residents.

As part of this study, we also aimed to evaluate the social factors or SDoH-related concerns that contributed to the persistent nonusers of video appointments. In our sample, the only factor associated with not using video appointments was not having a disability, handicap, or chronic disease. It is possible that their mobility or health allowed for greater flexibility in choosing an F2F visit or that they simply had fewer visits overall and were, therefore, less likely to choose video visits as an alternative. On the other hand, video appointments could be specifically beneficial for patients with disabilities or chronic illnesses who may have challenges with physical energy or mobility, be at higher risk for contracting illness when in public or have more health care appointments to attend overall. Given that there was limited variation in SDoH within our sample, this may have limited our ability to identify potential correlations between SDoH factors and selecting video or F2F appointments. Large-scale studies with socially diverse patients are required to fully understand the extent to which SDoH factors play in patients’ decision-making in choosing health care delivery methods. This understanding will further enhance patient outreach efforts and strengthen high-impact population health and research initiatives.

Since the pandemic, a significant public effort has been made to increase patients’ digital access at state, federal, and institutional levels [33]. Still, some patients may be unenthusiastic about attending video appointments [14,34]. We found that persistent nonusers of video appointments feel that they may not be able to “express” their concerns and are not able to “feel comfortable enough to talk openly” when having video appointments with primary care and psychiatry practicing physicians. Enjoying F2F encounters better than video appointments and potentially being unable to hear doctors clearly during a video appointment were 2 other beliefs that persistent nonusers of video appointments cited in high proportion. These patients conveyed these beliefs despite evidence of never engaging in video appointments in our institution. It is possible that individuals who consistently do not use video appointments have formed their opinions based on information obtained from sources other than their personal experience. These sources may include internet forums or the opinions of their peers. Another potential explanation could be poor digital experience when they attempted to engage in video appointments due to limited digital access (low-speed internet), language barrier, and low digital and health literacy [35]. It has been widely understood that poor digital experience could trigger patients’ anxiety regarding existing and emerging technology used in health care and may lead to its avoidance. This problem could be solved by appointing a “digital health coordinator” at the institutional level whose sole responsibility should be assisting patients with digital health solutions. This could overcome the perceived reluctance of patients to use digital services for health consultations. In addition, health care institutions should take into account the strong negative attitudes of this group toward video appointments when introducing them to digital technology for health care delivery.

Overall, our study results may inspire researchers to initiate a conversation about video adoption that goes beyond digital access and literacy. Our research examined the impact of SDoH and confidence or belief in video appointments adoption. Previous studies have not investigated which SDoH is most closely associated with video use. Furthermore, individuals with digital access and digital literacy may still choose to refrain from using video appointments. Therefore, objective measures should consider patient beliefs. Health care institutions should assess and evaluate patient preferences when implementing digital health care, especially those with digital competencies. We have yet to identify any digital literacy (validated) scales that have assessed an individual’s digital belief as one of the variables (negative vs positive) to assess overall digital literacy. From the perspective of behavior change theories [36], it is widely accepted that targeting one’s beliefs is essential for behavior change (digital adoption in this case). Therefore, our study adds novelty to the literature by informing researchers about understanding digital beliefs as a confounder in digital literacy and adoption. We suggest that through the community-based participatory research (CBPR) approach, researchers should attempt to identify facilitators to expedite behavior change. In a subsequent study with a larger sample size, it would be worth exploring if patients with limited interest in video appointments have sufficient digital access and literacy.

### Limitations

Our study has several limitations, including the low survey response rate, which may have led to selection bias, resulting in a study population that does not accurately represent the target population, and respondents may differ systematically from nonrespondents. We used the self-reported data and the possibility of recall bias. To help alleviate such a concern, we did verify eligibility and the existence of an F2F appointment via EHR. Additionally, though we tried to enroll participants from diverse backgrounds, the majority of patients in our sample were White, lived in urban areas, and did not experience major social challenges, limiting the generalizability of our findings. Our demographic variables were not extensive due to lack of availability or missing values in the data extracted from EHR. In addition, the survey did not include factors related to the health care system, such as whether patients requested a video appointment, if video appointments were encouraged and offered to patients, or if video appointments were offered but declined by the patient. The results of our study may also lack generalizability because the sample was derived from Mayo Clinic patients and there was no feasible way to assess if patients sought care outside Mayo Clinic and used video visits. However, we enrolled patients who have their primary care providers (PCPs) at Mayo Clinic (ie, paneled patients), reducing the likelihood of video visits being done outside of our health care system. According to FAIR Health [37], a national database of private and Medicare claims data, only 0.1% of all claims nationally in 2019 were related to telehealth. This percentage was even lower in rural areas. Based on these data, it is highly unlikely for patients with a PCP at Mayo Clinic to seek outside video-based care. It’s important to note that FAIR Health data include not just video visits, but also other telehealth technologies such as mobile health, remote patient monitoring, and store and forward technologies. We aimed to gather diverse data by including Mayo Clinic, Arizona, and Mayo Clinic, Florida. Despite a larger number of responses from Florida, the participants who responded were not from a diverse population. The studies show that there are differences in participation rates based on race, including lower rates of completing consent forms and responding to surveys, with Blacks and Hispanics being the most underrepresented [38]. Future work should explore the patterns of video use in more diverse patient populations, especially those who may be more likely to face barriers to health care (eg, patients living in rural areas and patients experiencing challenges with transportation). Our study also had several strengths, including that our sample was drawn from a multistate institution spanning rural and urban settings, the use of a validated measure of SDoH and the inclusion of scenario-based questions to better understand patients’ beliefs about video encounters.

### Conclusions

Our study identified sociodemographic factors and personal beliefs about video appointments that influenced patients’ decisions to choose between video versus F2F appointments for health care delivery. The patients who were older, lived in rural residences, sought care at Mayo Clinic Midwest, and who did not have access to the patient portal were more likely to be persistent nonusers of video appointments. We observed a single SDoH factor, that is not having a disability, handicap, or chronic diseases associated with persistent nonusers of video appointments. Not being able to adequately “express” their medical concerns and not “feel comfortable enough to talk openly” and adequately listen to their provider were notable beliefs held by persistent nonusers of video appointments. We recommend that health care institutions consider and evaluate patients’ strong negative beliefs toward video appointments when introducing them to digital technology for health care delivery. Conducting large-scale studies that encompass a diverse range of social and demographic backgrounds is imperative to comprehend why patients prefer video or in-person appointments. Only through such research can we identify the factors that influence their decision-making process.

## Appendix

Multimedia Appendix 1 Novel Strategies to Increase Telehealth Engagement (NSITE) Survey.

## Abbreviations

BB: broadband
EHR: electronic health record
F2F: face-to-face
MCHS: Mayo Clinic Health System
PCP: primary care provider
SDoH: social determinants of health
