# Supplementary material for: Demographics and Social Factors Associated With Persistent Nonuse of Video Appointments at a Multisite Health Care Institution: Cross-Sectional Study
Source: JMIR Form Res. 2024 Jan 24;8:e50572. doi: 10.2196/50572 (PMC10851122; doi:10.2196/50572)
Supplement: Multimedia Appendix 1 [file formative_v8i1e50572_app1.pdf]

MAYO
CLINIC

Novel Strategies to Increase Telehealth Engagement (NSITE)

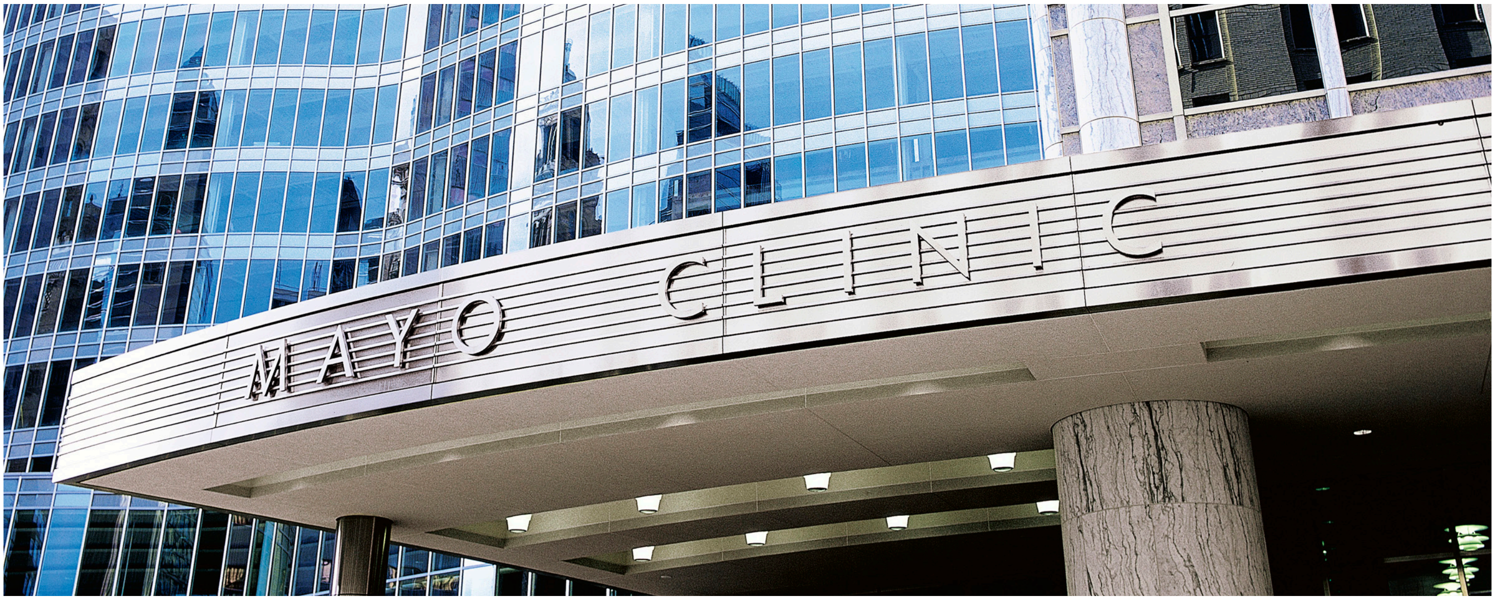

Survey Research Center

Clinic Number
Name
Address
Phone Number

Please enter above any missing information or change any that is incorrect.

The purpose of this survey is to learn about your experiences with video appointments, so we can improve patient care at Mayo Clinic.

Video appointments are a newer way to receive care. A video appointment is like an in-person appointment except that you are not in the same room with your healthcare provider. Instead, you use a computer or smart device to see and talk with them remotely using special software or an app.

For a video appointment to work you need:

- **An internet connection**
- **A desktop computer, laptop, tablet, or smartphone**
- **A camera connected to your device (like a webcam) or part of your device (like in some laptops, tablets, or smartphones)**
- **A microphone and a speaker OR a headset with built-in microphone**
- **A Patient Online Services account (also known as the Mayo Clinic Patient Portal)**

We would like to learn about your access to video equipment and software to do a video appointment.

INSTRUCTIONS: PLEASE CHECK THE APPROPRIATE BOX OR FILL IN THE BLANK AS INDICATED.

1. **Today's Date:** __ __/__ __/__ __ __ __
   Month Day Year

2. **Do you have access to broadband/high-speed internet for personal use?**

   1☐ No 2☐ Yes 3☐ Don't know

   If yes, do you have access to the internet in a place where you would feel comfortable having a video appointment with your doctor?

   1☐ No 2☐ Yes

3. **Do you have access to a device that can connect to the internet [e.g., cell phone, computer, tablet (e.g. iPad)]?**

   1 ☐ No 2 ☐ Yes

4. **Do you have a Mayo Clinic Patient Online Services account (Patient Portal)?**

   1 ☐ No 2 ☐ Yes 3 ☐ Don't know

5. **Do you ever need help using technology to have a video appointment?**

   1 ☐ No 2 ☐ Yes

   If yes, who do you primarily ask to help you with your video appointment?

   1 ☐ Family member
   2 ☐ Friend
   3 ☐ Neighbor
   4 ☐ Care provider (IE home health nurse, nursing assistant, etc.)

   How comfortable are you with having this person help you with your video appointment?

   1 ☐ Not at all comfortable
   2 ☐ A little comfortable
   3 ☐ Somewhat comfortable
   4 ☐ Very comfortable

6. **How confident do you feel in your ability to perform the following tasks?**

	Not at all confident	A little confident	Somewhat confident	Completely confident	Have never done this task
**Locating the camera or webcam on your device**	1 ☐	2 ☐	3 ☐	4 ☐	5 ☐
**Connecting to the internet**	1 ☐	2 ☐	3 ☐	4 ☐	5 ☐
**Using email**	1 ☐	2 ☐	3 ☐	4 ☐	5 ☐
**Finding the Mayo Clinic Patient Portal**	1 ☐	2 ☐	3 ☐	4 ☐	5 ☐
**Logging in to the Mayo Clinic Patient Portal**	1 ☐	2 ☐	3 ☐	4 ☐	5 ☐
**Checking for test results using the Mayo Clinic Patient Portal**	1 ☐	2 ☐	3 ☐	4 ☐	5 ☐
**Logging in to your video appointment using the Mayo Clinic Patient Portal**	1 ☐	2 ☐	3 ☐	4 ☐	5 ☐
**Sending a message to your doctor using the Mayo Clinic Patient Portal**	1 ☐	2 ☐	3 ☐	4 ☐	5 ☐

7. **How comfortable are you using technology to manage your health care online?** (For example, using patient online services to schedule appointments, check for test results, or send a message to your provider)

1 ☐ Not at all comfortable
2 ☐ A little comfortable
3 ☐ Somewhat comfortable
4 ☐ Very comfortable

We would like to learn about your experiences and opinions concerning health-care related video appointment.

8. **Have you ever had a video appointment with a healthcare provider?**

1 ☐ No   2 ☐ Yes

If yes, with what healthcare system was your most recent video appointment?

1 ☐ Mayo Clinic Rochester
2 ☐ Mayo Clinic Florida
3 ☐ Mayo Clinic Arizona
4 ☐ Mayo Clinic Health System
5 ☐ Other, not at Mayo Clinic

How would you rate your most recent video appointment experience?

1 ☐ Very good
2 ☐ Good
3 ☐ Acceptable
4 ☐ Poor
5 ☐ Very Poor

The next question asks about your experience with different medical specialties and which ones you think would or would not be well suited to video appointments.

9. **Which of the following medical specialties have you ever visited at Mayo Clinic? Please mark the specialty and indicate if it is well suited for a video appointment.** (Check all that apply.)

	**Well suited for a video appointment?** No	Yes	Not sure/ depends
1☐ Primary Care (Family Medicine/Internal Medicine)	1☐	2☐	3☐
1☐ Psychology and Psychiatry	1☐	2☐	3☐
1☐ Dermatology	1☐	2☐	3☐
1☐ Gynecology	1☐	2☐	3☐
1☐ Obstetrics	1☐	2☐	3☐
1☐ Neurology	1☐	2☐	3☐
1☐ Urology	1☐	2☐	3☐
1☐ Cardiology	1☐	2☐	3☐
1☐ Oncology	1☐	2☐	3☐
1☐ Gastroenterology	1☐	2☐	3☐
1☐ Surgery	1☐	2☐	3☐
1☐ Orthopedics	1☐	2☐	3☐
1☐ Sports Medicine	1☐	2☐	3☐
1☐ Physical Therapy	1☐	2☐	3☐
1☐ Other, please specify: ____________________	1☐	2☐	3☐

Scenario #1: Imagine you are having a video appointment with a <u>Mayo Clinic doctor for a general medicine health check-up</u> that ***does not require any procedures or exams*****. Further, imagine you have seen this doctor before for a face-to-face or in-person visit.**

10. **Please tell us how much you agree or disagree with the following statements regarding this scenario appointment being a video appointment rather than face-to-face.**

	Agree	Somewhat agree	Somewhat disagree	Disagree
**I am confident my doctor would be able to address any medical concerns effectively**	1☐	2☐	3☐	4☐
**I am confident I would be able to express all my concerns clearly**	1☐	2☐	3☐	4☐
**I am confident I would feel comfortable enough to talk openly**	1☐	2☐	3☐	4☐
**I feel video appointments should cost the same and are of equal value to face-to-face appointments**	1☐	2☐	3☐	4☐

Scenario #2: Imagine you are having an appointment with a Mayo Clinic Psychiatrist or Psychologist that *does not require any procedures or exams*. Further, imagine you have seen this doctor before for a face-to-face or in-person visit.

11. Please tell us how much you agree or disagree with the following statements regarding the appointment being a video appointment rather than face-to-face.

	Agree	Somewhat agree	Somewhat disagree	Disagree
**I am confident my doctor would be able to address any medical concerns effectively**	1☐	2☐	3☐	4☐
**I am confident I would be able to express all my concerns clearly**	1☐	2☐	3☐	4☐
**I am confident I would feel comfortable enough to talk openly**	1☐	2☐	3☐	4☐
**I feel video appointments should cost the same and are of equal value to face-to-face appointments**	1☐	2☐	3☐	4☐

12. How much do you agree or disagree with the following statements about the video appointments in general?

	Agree	Somewhat agree	Somewhat disagree	Disagree
**I am confident I would be able to understand when the doctor explains my symptoms/health condition**	1☐	2☐	3☐	4☐
**I am confident I would be able to read my doctor's facial expressions or non-verbal cues**	1☐	2☐	3☐	4☐
**I am confident I would be able to hear my doctor clearly**	1☐	2☐	3☐	4☐
**I would enjoy connecting with my doctor as much as if the appointment were face-to-face**	1☐	2☐	3☐	4☐
**I would feel comfortable talking with a doctor I have met before in-person**	1☐	2☐	3☐	4☐
**I would feel comfortable talking with a doctor I have never met before in-person**	1☐	2☐	3☐	4☐

13. If we provided information about video appointments, which topics should we cover? (Check all that apply.)

1 ☐ How to schedule/book a video appointment
1 ☐ How to download and use Zoom
1 ☐ How to locate and use your camera or webcam on your device
1 ☐ How to secure my personal internet connection
1 ☐ How to create a Patient Portal account
1 ☐ How to open a video appointment using the Patient Portal
1 ☐ How to send a message to your doctor using the Patient Portal
1 ☐ How to set up the space where you make the video meeting for the best video experience (clear sound, minimal background noise, good lighting)
1 ☐ How to set up the space where you make the video meeting to be private to protect your confidentiality from others who may be around you
1 ☐ How to find low-cost (or public) internet resources
1 ☐ How to find low-cost (or public) computer/tablet resources
1 ☐ How to help identify the medical conditions that likely can or cannot be best treated by video appointments
1 ☐ None of the above
1 ☐ Other, please specify: ____

14. What are your top 3 choices for receiving training/information about how to do a video appointment with your doctor?
(Check up to 3 choices.)

1 ☐ At places I visit in my community that are public (place of worship, government centers, community centers, parks, etc.)
1 ☐ E-mail
1 ☐ In-person
1 ☐ Mail/post
1 ☐ Phone
1 ☐ Website (mayoclinic.org)
1 ☐ Patient Portal App or website
1 ☐ Social media (e.g., Facebook, Instagram, Tiktok, Twitter, etc.)
1 ☐ Video conferences (e.g., Zoom, FaceTime, WhatsApp, etc.)
1 ☐ Other, please specify: ____

Now we would like to learn more about you. These questions are about your health and well-being, where you live, work, eat and sleep, as this information often can relate to your health care access.

15. How often do you need to have someone help you when you read instructions, pamphlets, or other written material from your doctor or pharmacy?

1 ☐ Never
2 ☐ Rarely
3 ☐ Sometimes
4 ☐ Often
5 ☐ Always

Which of the following information is accurate about you?

16. Food

	No	Yes	Prefer not to answer
**Within the past 12 months, did you worry that your food would run out before you got money to buy more?**	1 ☐	2 ☐	3 ☐
**Within the past 12 months, did the food you bought just not last, and you didn't have money to get more?**	1 ☐	2 ☐	3 ☐

17. Housing/Utilities

	No	Yes	Prefer not to answer
**Do you have housing?**	1 ☐	2 ☐	3 ☐
**Are you worried about losing your housing?**	1 ☐	2 ☐	3 ☐
**Within the past 12 months, have you or your family members you live with been without utilities, such as heat, electricity, or water, when it was really needed?**	1 ☐	2 ☐	3 ☐

18. Transportation

	No	Yes	Prefer not to answer
**Within the past 12 months, has lack of transportation kept you from medical appointments, getting your medicines, non-medical meetings or appointments, work, or from getting things that you need?**	1 ☐	2 ☐	3 ☐

19. Income

	No	Yes	Prefer not to answer
**Within the past 12 months, did you have trouble paying your bills?**	1 ☐	2 ☐	3 ☐

	No	Yes	Prefer not to answer
**20. Does any disability, handicap, or chronic disease make it difficult for you to engage in your typical day-to-day activities?**	1 ☐	2 ☐	3 ☐
**21. Are you currently working for pay?**	1 ☐	2 ☐	3 ☐

Thank you for completing this survey!

Please return your completed survey in the envelope provided.

If your envelope is missing, please mail your survey to:

Survey Research Center
Harwick 7
200 First Street SW
Rochester MN 55905

©2021 Mayo Foundation for Medical Education and Research. All rights reserved. MAYO, MAYO CLINIC and the triple-shield Mayo logo are trademarks and service marks of MFMER.

MC4269-000

(THIS PAGE IS FOR SURVEY RESEARCH TRACKING AND FILING PURPOSES ONLY)

Novel Strategies to Increase Telehealth Engagement (NSITE)

Investigator: Pravesh Sharma, M.D.

Version as of:
March 8, 2022 CAM
March 25, 2022
March 28, 2022
